# Experimental Validation of a Microwave System for Brain Stroke 3-D Imaging

**DOI:** 10.3390/diagnostics11071232

**Published:** 2021-07-08

**Authors:** David O. Rodriguez-Duarte, Jorge A. Tobon Vasquez, Rosa Scapaticci, Giovanna Turvani, Marta Cavagnaro, Mario R. Casu, Lorenzo Crocco, Francesca Vipiana

**Affiliations:** 1 Department of Electronics and Telecommunications, Politecnico di Torino, 10129 Torino, Italy; david.rodriguez@polito.it (D.O.R.-D.); jorge.tobon@polito.it (J.A.T.V.); giovanna.turvani@polito.it (G.T.); mario.casu@polito.it (M.R.C.); 2 Institute for the Electromagnetic Sensing of the Environment, National Research Council of Italy, 80124 Naples, Italy; scapaticci.r@irea.cnr.it (R.S.); crocco.l@irea.cnr.it (L.C.); 3 Department of Information Engineering, Electronics, and Telecommunications, Sapienza University of Rome, 00184 Roma, Italy; marta.cavagnaro@uniroma1.it

**Keywords:** microwave imaging, biomedical imaging, brain stroke monitoring, antenna array, hemorrhagic stroke, ischemic stroke

## Abstract

This paper experimentally validates the capability of a microwave prototype device to localize hemorrhages and ischemias within the brain as well as proposes an innovative calibration technique based on the measured data. In the reported experiments, a 3-D human-like head phantom is considered, where the brain is represented either with a homogeneous liquid mimicking brain dielectric properties or with ex vivo calf brains. The microwave imaging (MWI) system works at 1 GHz, and it is realized with a low-complexity architecture formed by an array of twenty-four printed monopole antennas. Each antenna is embedded into the “brick” of a semi-flexible dielectric matching medium, and it is positioned conformal to the head upper part. The imaging algorithm exploits a differential approach and provides 3-D images of the brain region. It employs the singular value decomposition of the discretized scattering operator obtained via accurate numerical models. The MWI system analysis shows promising reconstruction results and extends the device validation.

## 1. Introduction

Brain stroke is a medical emergency caused by the interruption of the regular supply of oxygen-rich blood to the brain, entailing the death of millions of brain cells per minute, and it can be the cause of death, disability, and dementia. The leading causes of stroke are blockage by a brain vessel clot or bleeding due to a vessel rupturing (or bursting). These different conditions cover 95% of the cases and are clinically termed ischemic stroke (IS) and intracranial hemorrhagic stroke (ICH), respectively [1].

Stroke aftercare relies widely on brain and vessel imaging technologies, identifying the specific pathophysiologic conditions for tailored treatment and enhancing effectiveness. Currently, computerized X-ray tomography (CT) and magnetic resonance imaging (MRI) are the most well-established imaging techniques, providing accurate and reliable images to clinicians [2]. However, MRI is costly and time-consuming, possibly causing a further delay in supplying proper therapy, and CT could be harmful due to the use of ionizing radiation. Moreover, these technologies are not feasible at the patient bedside and not always available at the first points of care, limiting their use in emergency situations and post-onset follow-up.

To overcome these limitations, microwave imaging (MWI) has emerged as a complementary imaging technique for brain stroke diagnosing and monitoring, albeit at the cost of a lower resolution. At microwave frequencies, brain tissues exhibit different dielectric properties that can allow their typology and physio-pathological status to be identified. For example, in ICH, severe internal bleeding causes an increment of the brain dielectric properties in the stroke-affected area. During an IS, instead, the reduction in the cerebral blood flow appears to decrease the dielectric properties of the ischemic zone, core, and penumbra [3,4]. To generate an image of the region of interest to be used as diagnostic information, MWI relies on the dielectric contrast that builds up between the background tissues and the pathological target one. In recent years, research groups have developed MWI prototype devices to detect, classify, and monitor brain strokes (see for example [5,6,7,8,9]). From the industrial side, two remarkable examples are the “Strokefinder” from Medfield Diagnostics, which is conceived to discriminate between ischemic and hemorrhagic strokes at an early stage, and the “BrainScanner” from EMTensor, which instead is capable of full brain tomographic imaging [10,11,12].

Here, we focus on the MWI prototype device recently introduced by the authors in [9], which is remarkable for its low-complexity architecture and the use of real-time imaging algorithms [13,14]. The proposed device has been designed for continuous monitoring of patients after stroke onset and during the first diagnosis thanks to its capability to detect localized brain tissue variations from measurements acquired at different times. In [9], the system was preliminarily tested using a homogeneous head phantom and a plastic spherical target to represent a stroke. However, plastic has a higher dielectric contrast within the brain with respect to intracranial hemorrhage and ischemia, impacting both the measured signal level and the imaging procedure. In this work, instead, the stroke is modeled using liquid mixtures mimicking both hemorrhagic and ischemic conditions. In addition, the brain tissues within the 3-D head phantom are either modeled using a liquid with the dielectric properties of an avergage human brain or assembled from ex vivo calf brains, bringing them closer to actual conditions (e.g., tissue inhomogeneity and complexity). Moreover, a novel measurement-based data calibration technique is proposed and experimentally verified. The obtained reconstructed images of the brain are then discussed, showing the capabilities of the MWI device in localizing the dielectric contrast variation between different stages.

The paper is organized as follows. Section 2 offers a comprehensive description of the components and functioning of the employed MWI system as well as of the preparation of the 3-D head phantoms. It also discusses the theoretical fundamentals on the imaging algorithm and the proposed calibration technique. Section 3 reports the reconstructed brain images obtained when processing the measured data, comprising hemorrhagic and ischemic conditions and different head phantoms, as well as the validation test of the procedure. Finally, Section 5 discusses the results and their implications, highlighting future research directions.

## 2. Materials and Methods

### 2.1. The Microwave Imaging Prototype Device

**Figure 1:**
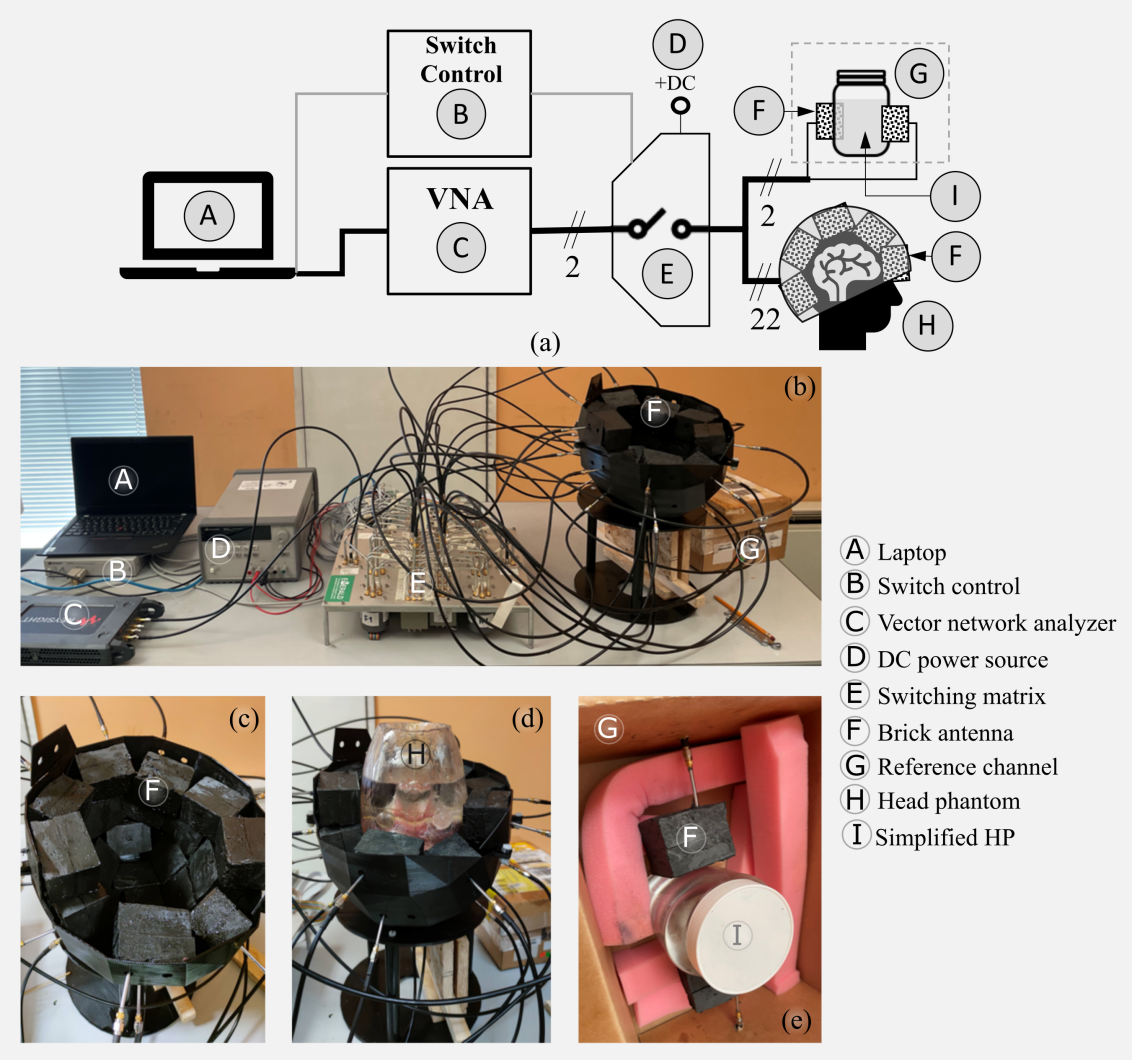
Microwave brain imaging system prototype: (**a**) block diagram, (**b**) overview, (**c**) 22-element antenna array helmet, (**d**) anthropomorphic adult head phantom within the helmet, and (**e**) reference channel components.

The prototype device comprises three main hardware parts: sensors/antennas, the transmitter/receiver (TX/RX) section, and signal routing. The block diagram that identifies the different parts of the MWI device is depicted in Figure 1a, together with the system overview in Figure 1b.

The sensors are simple and modular antennas with a “brick” shape (see Figure 1c) that integrates a monopole antenna, representing the radiating part, with a brick of dielectric material, acting as a matching medium [15]. The monopoles are fabricated using printed-circuit technology, guaranteeing repeatability and low-cost, and consist of a triangular-shaped element fed by a microstrip line connected to a rigid coaxial cable. The dielectric material around the monopole works as a matching medium, improving the electromagnetic (EM) signal penetration into the patient (or phantom) head. The material of the matching medium is a mixture of urethane rubber and graphite powder [16], which guarantees both the required dielectric properties and a degree of flexibility. The use of a solid yet flexible material simplifies the arrangement of the bricks in a helmet-like solution (see Figure 1c,d), which would not be possible with the more commonly used matching liquids. Moreover, the antennas are placed conformally on the head using a non-symmetrical and non-canonical distribution, to completely cover the brain area. However, the main directional component of most of the antennas is aligned horizontally within the transverse plane, while the distances between brick elements vary in the order of millimeters, i.e., in a range of 2–10 mm.

Following the design technique detailed in [13], the working frequency band is 0.8–1.2 GHz (here, the system works at 1 GHz), and the relative permittivity and conductivity of the matching material are $\epsilon_{r}=18.3$ and σ=0.19 S/m, respectively. The system includes 24 antennas, 22 of which are conformal to the upper part of the head while 2 are used for the reference channel, as shown in Figure 1e. The reference channel measures the transmission coefficient between the two antennas facing each other in a reference scenario (i.e., without the stroke) and is part of the calibration procedure (see Section 2.4). The reference channel was made with two antennas of the same kind as the ones placed around the head phantom. These two antennas are close to the MWI system and are connected to the same VNA through a dedicated path of the switching matrix in order to experience the same environmental conditions. Moreover, the two antennas of the reference channel are placed on the two sides of a cylindrical container filled with a liquid mimicking the dielectric properties of an average brain [17] to emulate the signal attenuation measured by the MWI system.

For transmission and reception, we uses the 2-port P9375A Keysight Streamline USB Vector Network Analyzer (VNA) [18], which is a compact yet highly precise VNA. We set the input power to 6 dBm and the intermediate filter (IF) to 10 Hz, which lowers the noise floor, hence providing adequate sensitivity to acquire the weak signals related to the EM field scattered by the imaged region (i.e., the stroke). The scattering parameters, measured at different time instants, were then used as input data of the imaging algorithm (as detailed in Section 2.3). Moreover, the VNA was used together with the 85070D Keysight dielectric probe to measure the phantom materials’ dielectric properties.

The role of the two-port VNA is to generate and collect signals for the 24 antennas around the head and in the reference channel, as a multi-view system requires. This is possible thanks to a custom-designed 2×24 switching matrix, which allows us to sequentially connect the VNA TX and RX ports to all of the antenna pairs. The switching matrix combines different high-quality electromechanical coaxial switches, which were selected for their minimum insertion loss and maximum isolation. The set of switches comprises two single-pole-four-throw (SP4T), eight single-pole-six-throw (SP6T), and twenty-four single-pole-double-throw (SPDT) switches. Connections between switches were made with semi-rigid coaxial cables, while flexible coaxial cables were used for the connections from the SPDT switches to the antennas [19]. A laptop controlled the entire measurement process and the signal acquisition via connections to the VNA and an electronic board controlling the switching matrix. The time for complete measurement set acquisition is around 6 min.

### 2.2. The Head and Stroke Phantoms

The head phantom, used in the following experiments, consisted of an anthropomorphic 3-D printed plastic container, while the brain was modeled using two different approaches.

The head container was made of clear resin (polyester casting resin) with dielectric properties similar to plastic and thickness around 3 mm. Hence, it could be compared electromagnetically with an air gap between the face of the brick antenna and the head surface. This constrain was studied by the authors in [15], where a brick antenna was placed at different distances from the phantom surface, showing adequate penetration in the range of interest for both homogeneous and multi-tissue head. Considering the air-gap limited effect on the signal penetration, the numerical model, used here to generate the discretized scattering operator, considers the head phantom container without thickness.

**Figure 2:**
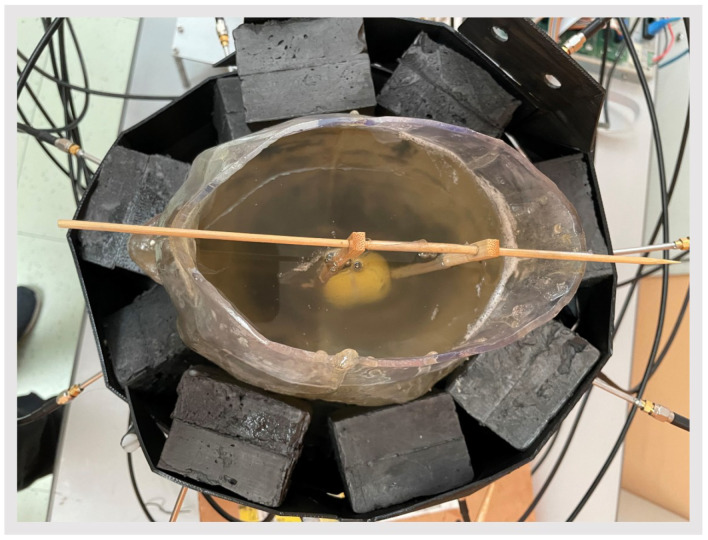
Homogeneous phantom (HP) with immersed stroke target and supporting wooden sticks.

Following an incremental methodology that moves from a simple scenario to a more complex one, initially, the brain was modeled with a liquid made of 38% Triton X-100, 62% water (volume percentages), and 5.2 g/L of salt, which mimics the average brain dielectric properties [17]. As shown in Figure 2, the whole head is filled with this mixture, which is also used in the reference channel (see Section 2.1). In the following, this model is called “homogeneous phantom” (HP).

**Figure 3:**
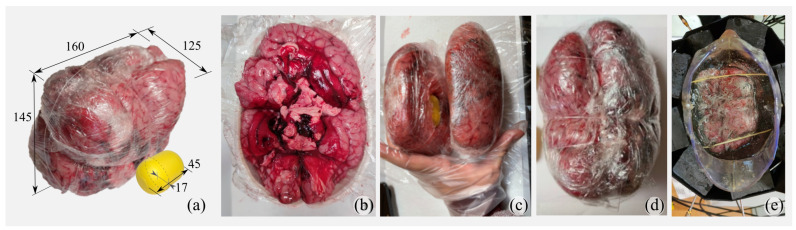
Multi-tissue phantom (MTP): (**a**) brain and stroke phantom (dimensions in mm), (**b**) one hemisphere preparation, (**c**) two hemispheres with inserted stroke, (**d**) joined brain, and (**e**) head phantom with immersed brain and filled up with electrically fat-mimicking liquid.

In the second, more complex scenario, the anthropomorphic plastic container was filled with a liquid mimicking the dielectric properties of human fat and a brain phantom made of calf brains. The fat-mimicking liquid was Triton X-100 [17,20]. The resultant brain phantom is shown in Figure 3a (The used calf brains were already available in the regular food distribution market, and the authors had no relation to or influence on the butchering.). Each hemisphere of the human adult brain was reproduced with two calf brains and shaped using a plastic 3-D printed shell, as shown in Figure 3b. The brain shell was used just to mold them as a human one and is not included in the measurement setup. The obtained hemisphere was, then, covered with plastic film (see Figure 3c) and tied together with a monofilament polymer line (see Figure 3d). The mass of the obtained brain was around 1.5 kg, with a volume of approximately $1200\;{\mathrm{cm}}^{3}$. Finally, the whole brain was inserted in the head plastic phantom, already filled with the liquid mimicking the fat. To prevent the brain from floating, it was fixed using a pair of wooden sticks, as shown in Figure 3e. This head model is referred to as a “multi-tissue phantom” (MTP) in the following.

**Figure 4:**
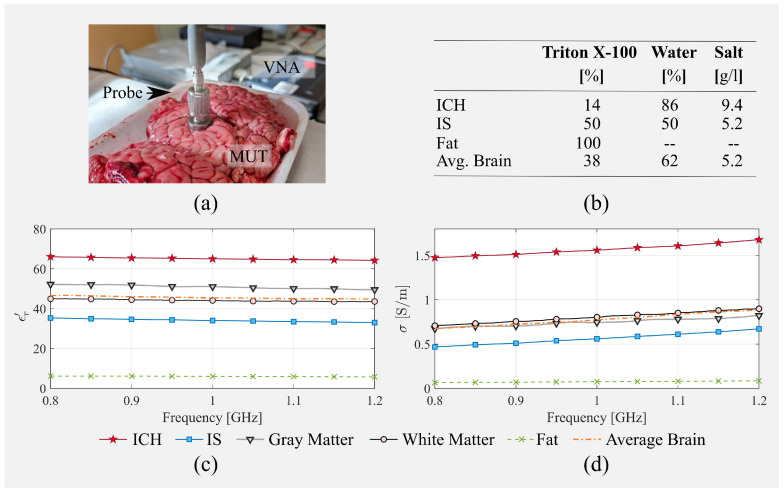
Dielectric properties characterization: (**a**) experimental setup, (**b**) tissue-mimicking liquid composition, (**c**) relative permittivity, and (**d**) conductivity.

The determination of the typical dimension of strokes (or their growth in volume) is far from trivial. The size of a stroke commonly ranges from about 2 to 200 ${\mathrm{cm}}^{3}$ depending on many factors such as the location, the interested blood vessels, and the time of diagnosis (see, e.g., [21,22,23,24]). The stroke tends to grow rapidly in the first hours after its onset, and clinical studies show that, after 72 h (rarely more), a stroke can still evolve, causing worsening of the patient’s condition. On the other hand, since the early follow-up of a stroke is currently based on clinical examinations and not on image-based diagnostics, there is no clear benchmark for stroke spread during the first hours after its onset. Here, the stroke is simulated using a very thin, less than a 1 mm, capsule-shaped plastic container filled with a liquid mimicking hemorrhagic or ischemic tissue (see the yellow capsule in Figure 3a), with a volume of around 50 ${\mathrm{cm}}^{3}$ that is in line with previously described findings. To fill-up the capsule, it is first sealed and then stuffed using a syringe. The stroke liquid is a mixture of Triton X-100, water, and salt. The ICH recipe is 14% Triton X-100, 86% water, and 9.4 g/L of salt, as reported in [17,20]. The IS recipe, on the contrary, was developed specifically for this work as 50% Triton X-100, 50% water, and 5.2 g/L of salt in order to obtain the dielectric properties in [3]. All of the recipes are summarized in Figure 4b.

The dielectric properties of tissues and liquids were measured using the open probe technique. The measurement system is composed of the two-port P9375 VNA, a low-phase coaxial cable, and the 85070D dielectric probe. The VNA measures the reflection coefficient of the probe, which is immersed (as in the case of liquids) or in direct contact (as in the case of the calf brain) with the material under test (MUT) and which depends on the unknown dielectric properties of the MUT. The unknown MUT dielectric properties can be obtained from the measured reflection coefficient via a modal expansion at the probe tip, quasi-static approaches, or lumped circuit models. In this work, we used the lumped element model proposed in [25], which was proven to be accurate [26]. Before the MUT measurement, a standard calibration procedure was performed. To this end, the probe reflection coefficient was measured in three known cases: air (open condition), closed on a perfect electric conductor (short), and inserted into distilled water (well-known load) [27]. The measured dielectric properties and the experimental brain characterization set up are shown in Figure 4. The characterization procedure of the calf-made brain considers white and gray matter separately.

### 2.3. The Imaging Algorithm

The imaging algorithm used in this work aims to recover a qualitative description of the variation in time of a brain pathology through 3-D images as required to monitor the stroke evolution. As input, it uses the scattering parameters measured by the MWI prototype described in Section 2.1, and the electric field obtained from full-wave simulation of a high-realistic twin model of system utilized to generate the imaging operator. The twin model mimics nominal system geometries and dielectric characteristics, including detailed antenna and phantoms models [9,15]. More formally, the algorithm attempts to reconstruct the temporal variation of the brain dielectric contrast, defined as

(1) $$\Delta \chi \left(r;t_{0},t_{1}\right)=\frac{\epsilon \left(r,t_{1}\right)-\epsilon \left(r,t_{0}\right)}{\epsilon_{b}\left(r\right)},$$

where $\epsilon (r,t_{0})$ and $\epsilon (r,t_{1})$ are the complex permittivities at the point r of the domain of interest *D* (i.e., the brain), at instants $t_{0}$ and $t_{1}$, respectively, while $\epsilon_{b}\left(r\right)$ is the complex permittivity of the reference or background scenario (i.e., the brain tissues without the presence of a stroke). The contrast Δχ is related to the *differential* scattering matrix ΔS, measured at the antenna ports, that is the difference between the scattering matrices measured at $t_{0}$ and $t_{1}$ time instants, respectively. The generic entry p,q of this matrix can be written as

(2) $$\Delta S\left(r_{p},r_{q};t_{0},t_{1}\right)=-\frac{j\;\omega \;\epsilon_{b}}{2\;a_{p}\;a_{q}}\int_{D}E\left(r_{p},r,t_{0}\right)\cdot E\left(r_{q},r,t_{1}\right)\;\Delta \chi \left(r,t_{0},t_{1}\right)\;dr,$$

where the symbol “·” denotes the dot product between vectors, *j* is the imaginary unit, ω=2πf is the angular frequency, and $a_{p}$ and $a_{q}$ are the known incoming root-power waves at the *p* and *q* antenna ports, respectively [28,29]. $E(r_{p},r,t_{0})$ and $E(r_{q},r,t_{1})$ are the electric fields radiated in *D* by the antenna in $r_{p}$ at $t_{0}$ and by the antenna in $r_{q}$ at $t_{1}$, respectively. Then, considering the localized time evolution of the stroke, the underlying inverse scattering problem takes advantage of the Born approximation, and the electric field radiated at $t_{1}$ is set equal to the initial one radiated at $t_{0}$ (assumed known from a diagnostic image previously collected), i.e., $E\left(r_{q},r,t_{1}\right)=E\left(r_{q},r,t_{0}\right)$. Thus, Equation (2) can be expressed in a compact form as

(3) $$\Delta S\left(r_{p},r_{q};t_{0},t_{1}\right)=S\left\{\Delta \chi \right\},$$

where S is now a linear and compact integral operator. Finally, Equation (2) can be inverted using the truncated singular value decomposition (TSVD) scheme [30], and the unknown dielectric contrast function is obtained as

(4) $$\Delta \chi =\sum_{n=1}^{T}\frac{1}{\sigma_{n}}\langle \Delta S,u_{n}\rangle v_{n},$$

where $\langle u,\sigma ,v\rangle$ is the SVD of the discretized scattering operator S and *T* is the truncation index that acts as a regularization parameter.

### 2.4. The Calibration Procedure

As mentioned in Section 2.3, the scattering matrices measured at different time instants are a primary input data of the imaging algorithm, and their accuracy determines the quality of the obtained reconstructions. However, in real scenarios, the input data might be corrupted by disturbances that degrade the system overall performance. Here, we present two calibration techniques to mitigate the unwanted measured data degradation, hence improving the reconstructed images, as shown later in Section 3.

The first proposed calibration scheme is termed in the following as “hardware calibration” (Hw), and it is based on the transmission coefficients measured in the reference channel described in Section 2.1. The Hw calibration aims to compensate for multiplicative inaccuracies on scattering transfer parameters between all the antenna pairs related to measurement uncertainties and performance changes due to environmental conditions (as e.g., room temperature variations) or drift errors in the VNA and/or in the switching matrix As detailed in Section 2.3, the measured input data of the imaging algorithm are the differential scattering parameters for each antenna pair $\Delta S(r_{p},r_{q};t_{0},t_{1})$, indicated in the following simply as $\Delta S_{p,q}\left(t_{0},t_{1}\right)$, where it is expected that the change between measurements at $t_{0}$ and $t_{1}$ is due to the scenario under test only, i.e., the evolution of the stroke area within the brain. *Unwanted* additional variations due to changes over time of the measurement conditions can significantly corrupt the measured differential data, possibly hiding the variation *searched* for in the image reconstruction.

To analyze this issue, we can write the differential scattering parameter for a generic antenna pair as

(5) $$\Delta S_{p,q}\;\left(t_{0},t_{1}\right)=S_{p,q}\left(t_{0}\right)-S_{p,q}\left(t_{1}\right)=\alpha \;{\bar{S}}_{p,q}\left(t_{0}\right)-\beta \;{\bar{S}}_{p,q}\left(t_{1}\right),$$

where $S_{p,q}\left(t_{0}\right)$ and $S_{p,q}\left(t_{1}\right)$ are the measured scattering parameters between the two considered antennas *p* and *q* at the two different time instants $t_{0}$ and $t_{1}$, while ${\bar{S}}_{p,q}\left(t_{0}\right)$ and ${\bar{S}}_{p,q}\left(t_{1}\right)$ are the corresponding scattering parameters in an ideal measurement scenario without inaccuracies, i.e., the ideal input data for the imaging algorithm. Finally, α and β are (unknown) complex numbers that model the possible multiplicative inaccuracies in the measured data at time instants $t_{0}$ and $t_{1}$, respectively, with respect to the ideal ones. Then, using the same notation, the transmission coefficient in the reference channel, measured in the same time instants $t_{0}$ and $t_{1}$, can be written as $S^{\mathrm{REF}}\left(t_{0}\right)=\alpha \;{\bar{S}}^{\mathrm{REF}}\left(t_{0}\right)$ and $S^{\mathrm{REF}}\left(t_{1}\right)=\beta \;{\bar{S}}^{\mathrm{REF}}\left(t_{1}\right)$, where we know that ${\bar{S}}^{\mathrm{REF}}\left(t_{0}\right)={\bar{S}}^{\mathrm{REF}}\left(t_{1}\right)$ because the scenario under test does *not* change here. Hence, the calibrated differential scattering parameters, ${\bar{\Delta S}}_{p,q}\;\left(t_{0},t_{1}\right)$, correspond to the difference in the ideal corresponding scattering parameters as follows:

(6) $$\begin{array}{ccc} {\bar{\Delta S}}_{p,q}\;\left(t_{0},t_{1}\right) & = & {\bar{S}}_{p,q}\left(t_{0}\right)-{\bar{S}}_{p,q}\left(t_{1}\right)\\ & = & \frac{1}{\alpha }\;\left[\alpha \;{\bar{S}}_{p,q}\left(t_{0}\right)-\frac{\alpha }{\beta }\;\beta \;{\bar{S}}_{p,q}\left(t_{1}\right)\right]\\ & = & \frac{1}{\alpha }\;\left[S_{p,q}\left(t_{0}\right)-\frac{S^{\mathrm{REF}}\left(t_{0}\right)}{S^{\mathrm{REF}}\left(t_{1}\right)}\;S_{p,q}\left(t_{1}\right)\right] \end{array}$$

where the multiplication factor 1/α can be neglected because it is in common for all the antenna pairs.

In addition to the described Hw calibration, a second calibration, dubbed in the following as “software calibration” (Sw), can be also applied. Its aim is to *tune* the measured scattering parameters with the system numerical modeling [31] used to generate the discretized scattering operator in Equation (4). The basic idea is to consider the same known scenario in both the numerical modeling and the real system [32]. The scattering data, measured and simulated in the known scenario, are then used to calibrate the scattering matrices, measured at different time instants in the scenario tested (which can be a real-world one). Here, we consider the head tested in healthy conditions, i.e., without stroke, as the known scenario. Hence, the hardware and software (Hw-Sw)-calibrated differential scattering parameters can be written as

(7) $${\tilde{\bar{\Delta S}}}_{p,q}\;\left(t_{0},t_{1}\right)=\frac{{\tilde{S}}_{p,q}^{H}}{S_{p,q}^{H}}\;{\bar{\Delta S}}_{p,q}\;\left(t_{0},t_{1}\right),$$

where ${\tilde{S}}_{p,q}^{H}$ and $S_{p,q}^{H}$ are the simulated and measured scattering parameters for the generic p,q antenna pair in the case of a healthy head, respectively.

## 3. Experimental Procedure and Results

The MWI system is validated via an experimental multi-stage procedure in the laboratory but mimicking realistic brain stroke scenarios. The test uses the experimental setup detailed in Section 2.1, the head and stroke phantoms described in Section 2.2, the differential imaging algorithm outlined in Section 2.3 applied to the measured data, and the calibration schemes outlined in Section 2.4.

The differential measuring scheme requires a consecutive pair of measurements at time instants denoted as $t_{0}$ and $t_{1}$ in Equation (1). In the following experimental testing, we consider the healthy scenario at $t_{0}$ (i.e., without the stroke) and a subsequent stroke-affected one at $t_{1}$. This setup is a simplified but equivalent experimental representation of the same variation in size of the stroke-affected brain region as it would be in a clinical scenario after stroke onset between time instants $t_{0}$ and $t_{1}$. Moreover, in both cases, the transmission coefficient is measured in the reference channel (see Figure 1d) in order to apply the Hw calibration (see Section 2.4). Hence, at $t_{0}$, the MWI system gathers a 22×22 S-matrix related to the healthy condition, which corresponds to the HP and MTP models described in Section 2.2. Then, at $t_{1}$, the stroke is introduced in the scenario. In the HP case, the stroke is immersed using a couple of wooden sticks as guidelines (see Figure 2). In the MTP case, the first measurement is performed on the brain within the brain-tissue-filled target. Then, the whole brain is extracted very carefully using fishing lines to pull each hemisphere out, one at a time, avoiding the maximum possible disturbances. Once the whole brain is out, it is cautiously untied so that the stroke target can be inserted into one of the hemispheres, and then, it is sealed and tied again. Finally, a new set of measurements is taken after positioning the brain back, this time with the stroke. Summarizing, in the HP case, it is sufficient to place the stroke using the guidelines, while for the MTP one, this is obtained by reintroducing the brain with the added stroke into the head.

To validate the MWI system performance, four study cases for each phantom model, including hemorrhagic and ischemic strokes located at various positions, are shown and discussed in the following.

**Figure 5:**
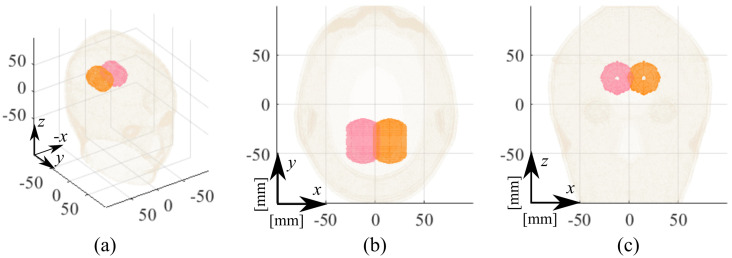
HP estimated stroke position in the experimental testing: I, pink capsule; II, orange capsule. (**a**) Three-dimensional view, (**b**) transverse view, and (**c**) frontal view.

In the HP case, we considered two different stroke locations within the brain region: around the occipital and temporal lobes. Those, labeled “I” and “II,” respectively, face each other and point to the inner face of the left and right brain hemispheres, as shown in Figure 5. Then, a pair of cases cover the ICH condition while the other two cover the IS one. The former pair is named “ICH-HP_I” and “ICH-HP_II”, and the latter “IS-HP_I” and “IS-HP_II”, respectively.

**Figure 6:**
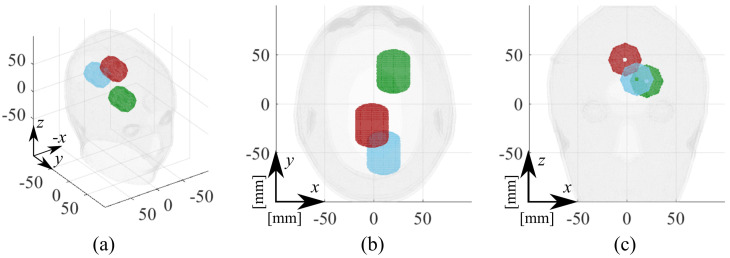
MTP estimated stroke positions in the experimental testing: 1, blue capsule; 2, red capsule; and 3, green capsule. (**a**) Three-dimensional view, (**b**) transverse view, and (**c**) frontal view.

The MTP is used with three different stroke locations, numbered in the following as 1, 2, and 3, while also covering both stroke typologies. The first and second cases, labeled as “ICH-MTP_1” and “IS-MTP_2”, respectively, consider hemorrhagic and ischemic strokes placed at two different locations. In the transverse view (i.e., from the top of the head), ICH-MTP_1 stroke is placed at the back of the head, mostly centered but slightly offset to the right. Instead, the IS-MTP_2 is more central than the previous one and slightly offset to the left as the capsule is placed in the other hemisphere. The last cases, labeled in the following as “ICH-MTP_3” and “IS-MTP_3”, share stroke locations (i.e., front left side) and study both typologies. A depth variation of around 2 cm is also considered between cases 1 and 2, where the former is slightly shallower than the latter, while the depth of case 3 is similar to that of case 1. Figure 6 illustrates the dimension and estimated stroke position of the considered cases with respect to the reference head. Finally, for better readability of the following analysis, Table 1 summarizes all of the considered cases, explaining the labels’ meanings.

**Figure 7:**
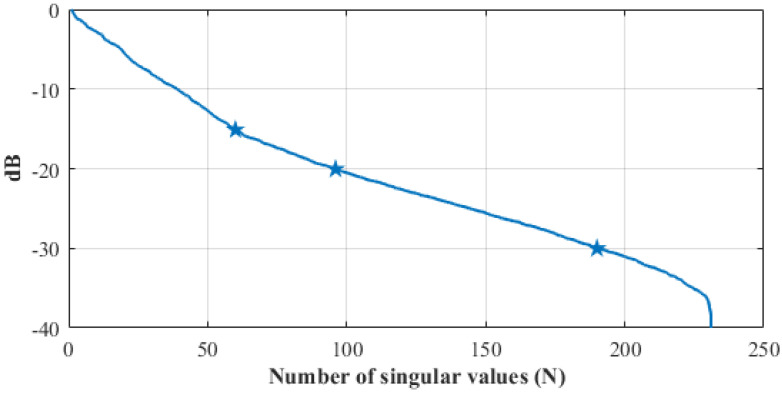
Singular values (dB) of the discretized scattering operator versus their indices; the stars indicate the three main slope changes.

Figure 7 shows the singular values of the discretized scattering operator (see Equation (4)), which was computed using the numerical model of the whole MWI system as in [9]. In the evaluation of the discretized scattering operator, the head was modeled as a homogeneous dielectric medium with the dielectric properties of the average brain. The reason for choosing this simplified head model is because all of the tissues’ details for the real head tested are in principle unknown once the MWI system is applied in a clinical scenario, and an average model can be used only, though images from other technologies might be used for building a different approximated operator. In Figure 7, the behavior of the singular values presents a sharp decrease around 240: this drop is due to the reciprocity of the measurement system, where for a generic p,q antenna pair, $S_{p,q}$ is equal to $S_{q,p}$.

The choice of the truncation index (i.e., *T* in Equation (4)) is a trade-off between accuracy and stability of the achievable reconstruction [30]. In principle, one would retain as many terms as possible in Equation (4) to retrieve the finer details of the unknown target. However, finer details correspond to singular values that are lower in magnitude, for which the contribution in the data is most likely overwhelmed by measurement noise and model errors. A simple and effective strategy to address the choice of *T* is to consider the changes in the slope of the singular values curve [30], as they correspond to a change in the singular functions involved in the image formation. In Figure 7, one can identify three main slope changes: the first one is at around −15 dB, the second one is at around −20 dB, and the last one is at around −30 dB. Considering the first threshold would lead to an over-regularized imaging solution, with poor resolution and information content. The last threshold, instead, would be affected by the uncertainties. To mediate between these two issue, the suitable choice for *T* (for the considered scattering operator) can be identified as the one corresponding to −21 dB (number of singular values equal to 104, which is used for all the following reconstruction).

**Figure 8:**
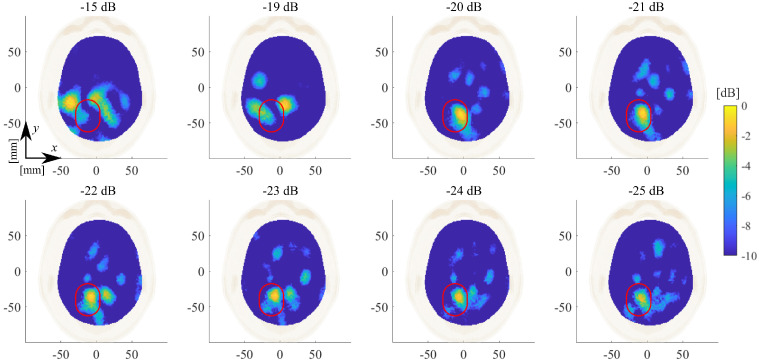
ICH-HP_I amplitude values of the normalized reconstructed dielectric contrast in the transverse plane view varying the threshold on the singular values between −15 and −25 dB. The expected stroke area is indicated with a red line.

**Figure 9:**
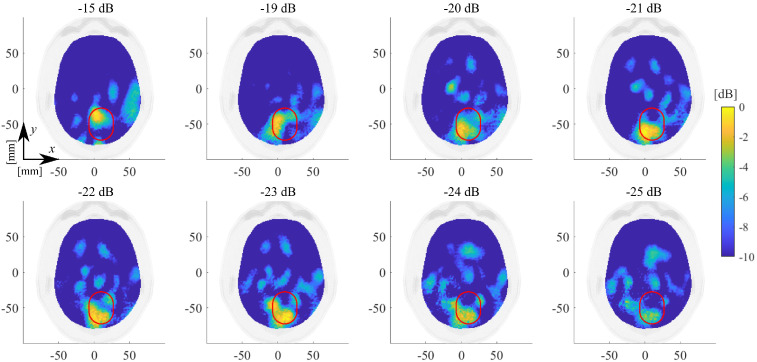
ICH-MTP_1 amplitude values of the normalized reconstructed dielectric contrast in the transverse plane view varying the threshold on the singular values between −15 and −25 dB. The expected stroke area is indicated with a red line.

Figure 8 and Figure 9 illustrate the normalized dielectric contrast as the threshold coefficient varies after applying the proposed Hw-Sw calibration for the ICH-HP_I and the ICH-MTP_1 cases, respectively.

**Figure 10:**
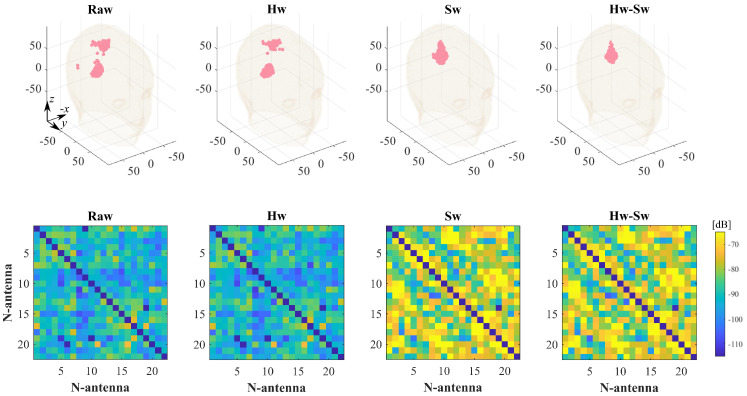
Calibration schemes applied to the ICH-HP_I case. Columns (from left to right): no calibration, Hw calibration, Sw calibration, and Hw and Sw calibrations. Top-row: normalized reconstructed dielectric contrast values above −3 dB (all the units are in mm); bottom-row: differential scattering matrices’ amplitude values in dB (the main diagonal is not shown).

**Figure 11:**
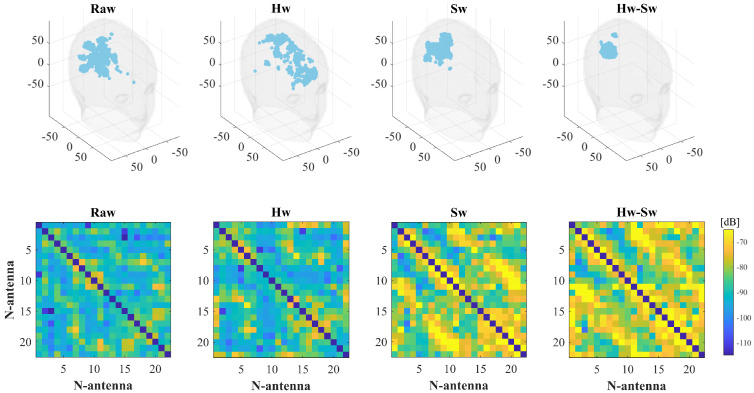
Calibration schemes applied to the ICH-MTP_1 case. Columns (from left to right): no calibration, Hw calibration, Sw calibration, and Hw and Sw calibrations. Top-row: normalized reconstructed dielectric contrast values above −3 dB (all the units are in mm); bottom-row: differential scattering matrices’ amplitude values in dB (the main diagonal is not shown).

For the same cases ICH-HP_I and ICH-MTP_1, Figure 10 and Figure 11 show the effect of applying the calibration procedures described in Section 2.4 to the measured data. For each data calibration procedure, the top row of Figure 10 and Figure 11 shows the corresponding normalized reconstructed dielectric contrast values above −3 dB, while the bottom one presents the amplitude values of the measured differential scattering matrices. Here, the differential matrix’s self-terms are not shown because they are not given in input as the imaging algorithm (they are highly dependent on the antenna’s manufacturing variability and are not modeled in the scattering operator) [9]. Moving through the columns of Figure 10 and Figure 11, the first contemplates the case without any calibration, labeled as “Raw”. Then, the last three columns cover the calibrated scenarios. The middle columns show where the hardware and software calibrations are applied independently, and the last one applies the hardware calibration first and then the software one. These columns are dubbed as “Hw”, “Sw”, and “Hw-Sw”, respectively.

**Figure 12:**
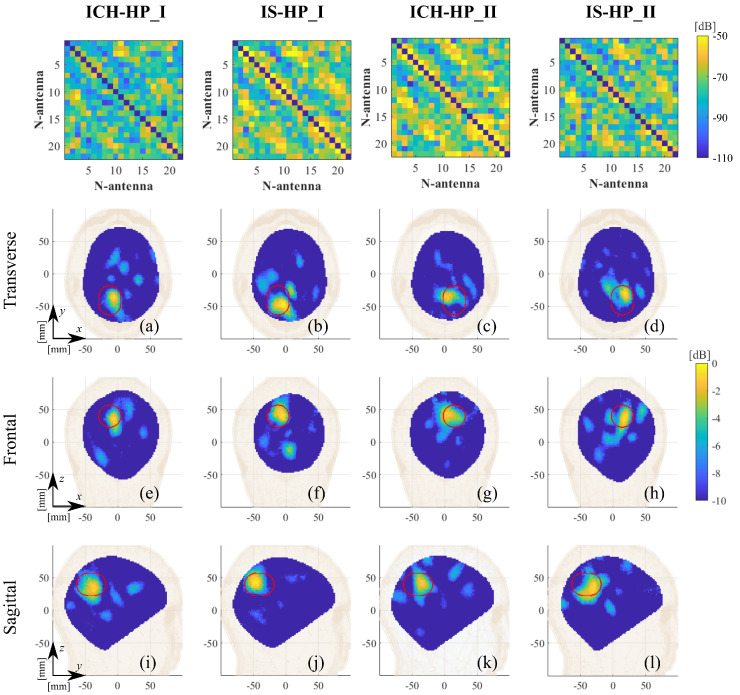
HP calibrated differential scatting matrices (first row) and reconstructed normalized dielectric contrast values. Columns (from left to right): ICH-HP_I, IS-HP_I, ICH-HP_II, and IS-HP_II. (**a**–**d**) Transverse views, (**e**–**h**) frontal views, and (**i**–**l**) sagittal views. The expected stroke area is indicated with a red line.

**Figure 13:**
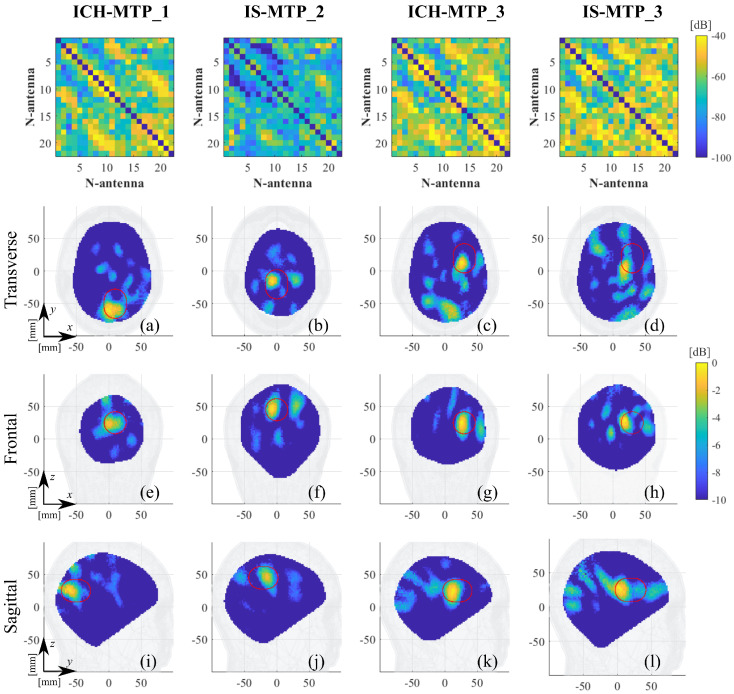
MTP calibrated differential scatting matrices (first row) and reconstructed normalized dielectric contrast values. Columns (from left to right): ICH-MTP_1, IS-MTP_2, ICH-MTP_3, and IS-MTP_3. (**a**–**d**) Transverse views, (**e**–**h**) frontal views, and (**i**–**l**) sagittal views. The expected stroke area is indicated with a red line.

In Figure 12 and Figure 13, we report the differential scattering matrices after calibrations (top row) and the reconstructed normalized dielectric contrast for all studied scenarios (see Table 1), showing the three main cross-sectional views (transverse, frontal, and sagittal) centered at its maximum. In all plots, the estimated stroke position, size, and orientation (see Figure 5 and Figure 6) are indicated with a red capsule shape. Moreover, to quantitatively evaluate the 3-D reconstructions obtained, the last column in Table 1 reports the root mean square error (RMSE), defined as

(8) $$RMSE=\sqrt{\frac{\sum_{n=1}^{N_{s}}{\left[\Delta \hat{\chi }\left(r_{n}\right)-\Delta \chi \left(r_{n}\right)\right]}^{2}}{N_{s}}}$$

where $N_{s}$ is the number of samples $r_{n}$ of the discretized domain, $\Delta \hat{\chi }$ the retrieved differential contrast, and Δχ is the actual contrast.

Finally, to validate the measurements, we performed false-positive and base tests. To assess the possible false-positive scenarios, the imaging reconstruction was tested using a differential scattering matrix of healthy cases only, obtained with both homogeneous and multi-tissue phantoms.

**Figure 14:**
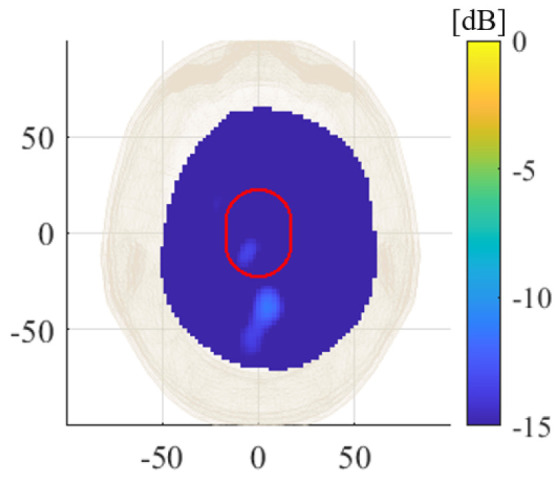
Amplitude values of the reconstructed dielectric contrast for a false-positive scenario in the HP case; transverse view cut in its maximum, normalized to the maximum value of ICH-HP_I. The red circle indicates the position of the average brain filled target.

In the case of the homogeneous phantom, to also evaluate the effect of the plastic shell, we considered the differential scattering matrix, measured with and without the presence of the plastic capsule filled with the average brain liquid in the head. The obtained reconstructed dielectric contrast, normalized to the maximum value of ICH-HP_I, is shown in Figure 14 (cross-cut in its maximum in the transverse plane). This result confirms that the effect of plastic container (highlighted with a red contour) is negligible.

**Figure 15:**
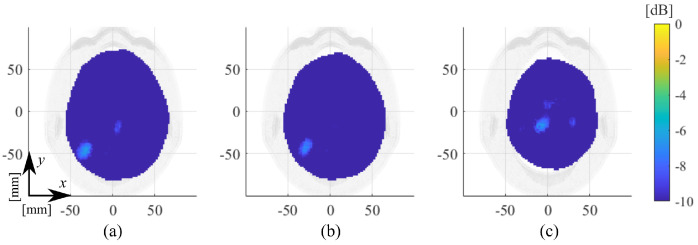
Amplitude values of the reconstructed dielectric contrast for false-positive scenarios in the case of different time intervals in the MTP case; transverse views cut in their maximum, normalized to the maximum value of ICH-MTP_1: (**a**) 35 min, (**b**) 1 h and 45 min, and (**c**) 1 h and 10 min.

Then, in the case of the multi-tissue phantom, we report three cases with different time-lapses between measurement sets. The first one, shown in Figure 15a, considers a measured setup with an unaltered scenario. In other words, the calf-made brain in the MTP is not removed between the measuring. This initial case presents a time distance of around 35 min between the two considered scattering matrix measurements. Instead, the last two cases regard a more challenging scenario where, between the two considered measurement sets, the brain was removed and re-introduced in the head phantom. In Figure 15b, the time distance between the two considered measurement sets is around 1 h and 45 min, while in Figure 15c, it is around 1 h and 10 min.

**Figure 16:**
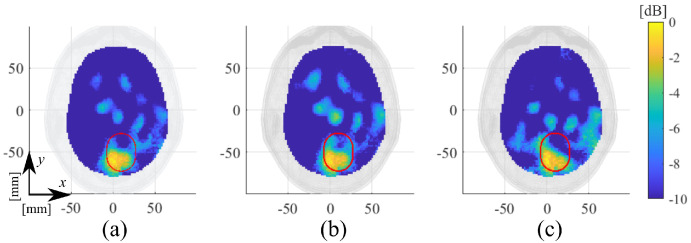
Amplitude values of the reconstructed dielectric contrast for re-test experiment; transverse views cut in their maximum, normalized to the maximum value of ICH-MTP_1: (**a**) baseline, (**b**), re-test 1, and (**c**) re-test 2.

To verify that different measurements of the same scenario generate the same reconstructed dielectric contrast, we considered four consecutive measurements, starting with one healthy scenario, followed by two ICH target measurements and finishing with another healthy one. As baseline, the first and second measurement sets are used to generate the reconstructed dielectric contrast in Figure 16a that corresponds to the ICH-MTP_1 case, also reported in Figure 13a. Then, the first re-test, shown in Figure 16b, is with the initial healthy scenario (i.e., first measurement set) and the second ICH target case (i.e., third measurement set). Instead, the second re-test, shown in Figure 16c, considers the second ICH target case (i.e., third measurement set) and the last healthy scenario (i.e., fourth measurement set). Comparing the reconstructed dielectric contrast, it is evident that the imaging algorithm is able to correctly recover the position of the stroke, reconstructing a similar shape and confirming the measurement’s repeatability.

## 4. Discussion

In the following, the selected algorithm parameters, the calibration procedures, and the obtained imaging results are commented upon and possible perspectives are discussed.

**Figure 17:**
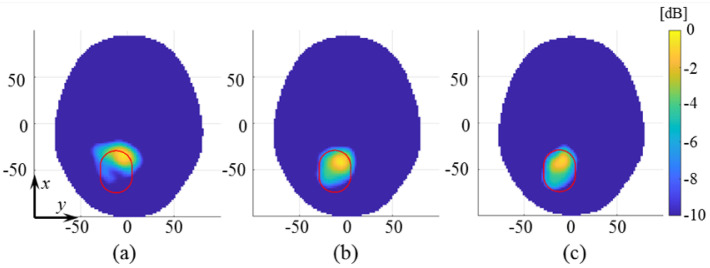
ICH-HP_I amplitude values of the normalized reconstructed dielectric contrast obtained with noiseless simulations and varying the threshold on the singular values: (**a**) −21 dB, (**b**) −30 dB, and (**c**) −40 dB.

As mentioned in Section 2.3, the imaging algorithm used here is the TSVD scheme. Thus, the singular value truncation threshold plays a key role in the achievable quality of the reconstructed normalized dielectric contrast, expecting better retrieved extension but stronger noisy detrimental effects as the threshold decreases. We chose a threshold equal to −21 dB as a trade-off between accuracy and stability, which allows for locating the stroke properly (see Figure 8, Figure 9, Figure 12 and Figure 13), and this is a significant clinical aspect but with a reconstructed shape that can be poor, missing some parts of the expected stroke extent (indicated with a red line in all figures). Hence, further numerical analyses are needed to better investigate this point. In this paper, the same experimental scenario shown in Figure 8 was numerically simulated using an in-house FEM-based EM solver [31], changing the chosen singular value truncation threshold. In Figure 17, the obtained normalized reconstructed dielectric contrast is shown for a singular value threshold equal to −21 dB (as chosen in the experiments), −30 dB, and −40 dB (i.e., the minimum one including all the singular values greater than zero). If the singular value threshold is equal to −21 dB (see Figure 17a), similarly to that with the experimental data, the imaging algorithm is able to locate the stroke position properly, but it misses part of its extent (shown in Figure 17 with a red line). Then, as expected, if the threshold decreases, the stroke extension is better reconstructed, as shown in Figure 17b,c. However, in the simulated scenario, the data are noiseless and the singular values threshold can be decreased without affecting the quality of the reconstructions. Instead, the experimental data are noisy, and hence, the singular values have to be truncated at such a level to guarantee the stability of the achieved reconstruction. As a matter of fact, to be able to reduce the singular values threshold, the noise of the measured data has to be reduced, e.g., implementing a more stable phantom (without the need to modify it at every measurement), having more accurate control of the antenna position and orientation with respect to the head phantom and, maybe, substituting the 2×24 switching matrix with a high-performance 24-ports VNA. These system improvements will be investigated in future research activities.

Regarding the proposed calibration techniques, the results in Section 3 show that both Hw and Sw calibrations, in general, help to improve in the stroke retrieve. Hence, we found that the best strategy is to apply both calibrations together (last column of Figure 10 and Figure 11), which delivers a more robust differential scattering matrix in input to the TSVD algorithm and, thus, improves the quality of 3-D reconstructed images. It is also worth mentioning that, when the raw reconstruction is already well defined, as expected, the calibration does not add significant enhancements, as in the ICH-HP_I case, where the Hw calibration has a minor effect.

To quantify the quality of the reconstructions, the RMSE is reported in the last column of Table 1. The average RMSE is around 0.2, with values slightly lower for HP cases with respect to MTP ones, as somehow expected for the additional disturbances in the MTP measuring process. These values agree with the findings of the measured plastic-stroke scenario in [9]. Although, they are still higher in comparison to noiseless and white-noise-affected simulated cased cases [9,31], leaving room for additional improvement through e.g., novel system-based calibration techniques [33].

Finally, several validation tests in Section 3 evaluated the system capabilities against false positive cases. In the first tests, shown in Figure 14 and Figure 15, four false-positive scenarios were investigated, one with the HP and three with the MTP, where for the latter two MTP cases, the brain was removed and re-introduced in the head phantom. The normalized reconstructed dielectric contrast values are always significantly lower than the corresponding ones when the stroke is present. These results verify the negligible effect of the plastic target shell and a certain grade of robustness of the MWI system to inaccuracies that could arise in a real-life scenario where the antennas positioning could change slightly, with respect the patient head, between different measurement sets. Lastly, the repetition test in Figure 16 shows how the MWI system delivers very close reconstruction results under a similar scenario, confirming good measurements repeatability

## 5. Conclusions

This work focused on the experimental assessment of the MWI system recently presented in [9] and assessed via numerical simulations in [31], showing that the system capabilities identify the presence of both intracranial hemorrhagic and ischemic strokes. The reported experiments have been performed in realistic scenarios, including human-like 3-D phantoms with the brain made of ex vivo calf brains and clinical-sized comparable hemorrhages and ischemias, mimicked using liquids. Moreover, novel calibration techniques based on measured data involving an auxiliary measurement reference channel have been proposed and validated to mitigate environmental and parametric changes over time. 

## Figures and Tables

**Table 1:** Description of all of the considered cases.

Label	Stroke	Phantom	Position	RMSE
ICH_HP-I	Hemorrhagic	Homogeneous	I, pink, Figure 5	0.17
ICH_HP-II	Hemorrhagic	Homogeneous	II, orange, Figure 5	0.19
IS_HP-I	Ischemic	Homogeneous	I, pink, Figure 5	0.22
IS_HP-II	Ischemic	Homogeneous	II, orange, Figure 5	0.21
ICH_MTP-1	Hemorrhagic	Multi-tissue	1, blue, Figure 6	0.20
ICH_MTP-3	Hemorrhagic	Multi-tissue	3, red, Figure 6	0.22
IS_MTP-2	Ischemic	Multi-tissue	2, green, Figure 6	0.21
IS_MTP-3	Ischemic	Multi-tissue	3, green, Figure 6	0.24

## Abbreviations

The following abbreviations are used in this manuscript:

CT	Computerized tomography
EM	Electromagnetic
HP	Homogeneous phantom
IF	Intermediate frequency
IHC	Intracranial hemorrhagic stroke
IS	Ischemic stroke
MRI	Magnetic resonance imaging
MTP	Multi-tissue phantom
MWI	Microwave imaging
RX	Receiver
SP4T	Single-pole-four-throw
SPDT	Single-pole-double-throw
TSVD	Truncated singular value decomposition
TX	Transmitter
VNA	Vector network analyzer

## References

[1] Benjamin E.J., Muntner P., Bittencourt M.S. (2019). Heart disease and stroke statistics-2019 update: A report from the American Heart Association. Circulation.

[2] Walsh K.B. (2019). Non-invasive sensor technology for prehospital stroke diagnosis: Current status and future directions. Int. J. Stroke.

[3] Semenov S.Y., Corfield D.R. (2008). Microwave tomography for brain imaging: Feasibility assessment for stroke detection. Int. J. Antennas Propagat..

[4] Semenov S., Huynh T., Williams T., Nicholson B., Vasilenko A. (2017). Dielectric properties of brain tissue in acute ischemic stroke: Experimental study on swine. Bioelectromagnetics.

[5] Fedeli A., Estatico C., Pastorino M., Randazzo A. (2020). Microwave Detection of Brain Injuries by Means of a Hybrid Imaging Method. IEEE Open J. Antennas Propag..

[6] Alqadami A.S.M., Nguyen-Trong N., Mohammed B., Stancombe A.E., Heitzmann M.T., Abbosh A. (2020). Compact Unidirectional Conformal Antenna Based on Flexible High-Permittivity Custom-Made Substrate for Wearable Wideband Electromagnetic Head Imaging System. IEEE Trans. Antennas Propag..

[7] Karadima O., Rahman M., Sotiriou J., Ghavami N., Lu P., Ahsan S., Kosmas P. (2020). Experimental Validation of Microwave Tomography with the DBIM-TwIST Algorithm for Brain Stroke Detection and Classification. Sensors.

[8] Merunka I., Massa A., Vrba D., Fiser O., Salucci M., Vrba J. (2019). Microwave Tomography System for Methodical Testing of Human Brain Stroke Detection Approaches. Int. J. Antennas Propag..

[9] Tobon Vasquez J.A., Scapaticci R., Turvani G., Bellizzi G., Rodriguez-Duarte D.O., Joachimowicz N., Duchêne B., Tedeschi E., Casu M.R., Crocco L. (2020). A Prototype Microwave System for 3D Brain Stroke Imaging. Sensors.

[10] Candefjord S., Winges J., Malik A., Yu Y., Rylander T., McKelvey T., Fhager A., Elam M., Persson M. (2017). Microwave technology for detecting traumatic intracranial bleedings: Tests on phantom of subdural hematoma and numerical simulations. Med. Biol. Eng. Comput..

[11] Fhager A., Candefjord S., Elam M., Persson M. (2018). Microwave Diagnostics Ahead: Saving Time and the Lives of Trauma and Stroke Patients. IEEE Microw. Mag..

[12] Hopfer M., Planas R., Hamidipour A., Henriksson T., Semenov S. (2017). Electromagnetic Tomography for Detection, Differentiation, and Monitoring of Brain Stroke: A Virtual Data and Human Head Phantom Study. IEEE Antennas Propag. Mag..

[13] Scapaticci R., Tobon J., Bellizzi G., Vipiana F., Crocco L. (2018). Design and Numerical Characterization of a Low-Complexity Microwave Device for Brain Stroke Monitoring. IEEE Trans. Antennas Propag..

[14] Sarwar I., Turvani G., Casu M.R., Tobon Vasquez J.A., Vipiana F., Scapaticci R., Crocco L. (2018). Low-Cost Low-Power Acceleration of a Microwave Imaging Algorithm for Brain Stroke Monitoring. J. Low Power Electron. Appl..

[15] Rodriguez-Duarte D.O., Vasquez J.A.T., Scapaticci R., Crocco L., Vipiana F. (2020). Brick-Shaped Antenna Module for Microwave Brain Imaging Systems. IEEE Antennas Wirel. Propag. Lett..

[16] Garrett J., Fear E. (2014). Stable and Flexible Materials to Mimic the Dielectric Properties of Human Soft Tissues. IEEE Antennas Wirel. Propag. Lett..

[17] Joachimowicz N., Duchêne B., Conessa C., Meyer O. (2018). Anthropomorphic Breast and Head Phantoms for Microwave Imaging. Diagnostics.

[18] Keysight Technologies (2018). Keysight Streamline Series USB Vector Network Analyzer P937XA 2-port, up to 26.5 GHz. Data Sheet Tech. Specif..

[19] Tobon Vasquez J.A., Scapaticci R., Turvani G., Bellizzi G., Joachimowicz N., Duchêne B., Tedeschi E., Casu M.R., Crocco L., Vipiana F. (2019). Design and Experimental Assessment of a 2D Microwave Imaging System for Brain Stroke Monitoring. Int. J. Antennas Propag..

[20] Joachimowicz N., Vasquez J.T., Turvani G., Dassano G., Casu M.R., Vipiana F., Duchêne B., Scapaticci R., Crocco L. Head Phantoms for a Microwave Imaging System Dedicated to Cerebrovascular Disease Monitoring. Proceedings of the 2018 IEEE Conference on Antenna Measurements Applications (CAMA).

[21] Laredo C., Zhao Y., Rudilosso S., Renu A., Pariente J.C., Chamorro A., Urra X. (2018). Prognostic Significance of Infarct Size and Location: The Case of Insular Stroke. Sci. Rep..

[22] Bruno A., Shah N., Akinwuntan A.E., Close B., Switzer J.A. (2013). Stroke size correlates with functional outcome on the simplified modified Rankin Scale questionnaire. J. Stroke Cerebrovasc. Dis..

[23] Saver J.L. (2006). Time Is Brain-Quantified. Stroke.

[24] Saver J.L., Johnston K.C., Homer D., Wityk R., Koroshetz W., Truskowski L.L., Haley E.C. (1999). Infarct Volume as a Surrogate or Auxiliary Outcome Measure in Ischemic Stroke Clinical Trials. Stroke.

[25] Stuchly M., Stuchly S. (1980). Coaxial line reflection method for measuring dielectric properties of biological substances at radio and microwave frequencies. IEEE Trans. Instrum. Meas..

[26] Cavagnaro M., Ruvio G. (2020). Numerical Sensitivity Analysis for Dielectric Characterization of Biological Samples by Open-Ended Probe Technique. Sensors.

[27] Kraszewski A., Stuchly M., Stuchly S. (1983). VNA calibration method for measurements of dielectric properties. IEEE Trans. Instrum. Meas..

[28] Tajik D., Foroutan F., Shumakov D.S., Pitcher A.D., Nikolova N.K. (2018). Real-Time Microwave Imaging of a Compressed Breast Phantom with Planar Scanning. IEEE J. Electromagn. RF Microw. Med. Biol..

[29] Beaverstone A.S., Shumakov D.S., Nikolova N.K. (2017). Frequency-Domain Integral Equations of Scattering for Complex Scalar Responses. IEEE Trans. Microw. Theory Technoques.

[30] Bertero M., Boccacci P. (1998). Introduction to Inverse Problems in Imaging.

[31] Rodriguez-Duarte D.O., Tobon Vasquez J.A., Scapaticci R., Crocco L., Vipiana F. (2021). Assessing a Microwave Imaging System for Brain Stroke Monitoring via High Fidelity Numerical Modelling. IEEE J. Electromagn. Microw. Med. Biol..

[32] Gilmore C., Mojabi P., Zakaria A., Ostadrahimi M., Kaye C., Noghanian S., Shafai L., Pistorius S., LoVetri J. (2010). A Wideband Microwave Tomography System with a Novel Frequency Selection Procedure. IEEE Trans. Biomed. Eng..

[33] Rodriguez-Duarte D.O., Tobon Vasquez J.A., Vipiana F. Hybrid Simulation-Measurement Calibration Technique for Microwave Imaging Systems. Proceedings of the 2021 European Conference on Antennas and Propagation (EuCAP).

